# Elevated expression of HSP90 and the antitumor effect of an HSP90 inhibitor via inactivation of the Akt/mTOR pathway in undifferentiated pleomorphic sarcoma

**DOI:** 10.1186/s12885-015-1830-8

**Published:** 2015-10-26

**Authors:** Hirofumi Bekki, Kenichi Kohashi, Akira Maekawa, Yuichi Yamada, Hidetaka Yamamoto, Katsumi Harimaya, Michiyuki Hakozaki, Kazuki Nabeshima, Yukihide Iwamoto, Yoshinao Oda

**Affiliations:** Department of Anatomic Pathology, Graduate School of Medical Sciences, Kyushu University, Maidashi 3-1-1, Higashi-ku, Fukuoka 812-8582 Japan; Orthopaedic Surgery, Graduate School of Medical Science, Kyushu University, Fukuoka, Japan; Department of Orthopaedic Surger, Fukushima Medical University School of Medicine, Fukushima, Japan; Department of Pathology, Graduate School of Medical Science, Fukuoka University, Fukuoka, Japan

**Keywords:** Undifferentiated pleomorphic sarcoma, Heat shock protein 90, Akt/mammalian target of rapamycin pathway, Mitogen-activated protein kinase pathway, Phosphorylation

## Abstract

**Background:**

Undifferentiated pleomorphic sarcoma (UPS) is a heterogeneous tumor group, and little is known about molecular target therapy for UPS. Heat shock protein 90 (HSP90) is an expressed chaperone that refolds certain denatured proteins under stress conditions. One of these proteins is Akt. The disruption of Akt signaling plays an important role in tumor progression. The present study’s purpose was to analyze the HSP90 expression, Akt/mTOR pathway activation and the correlation between HSP90 expression and its pathway activation in UPS.

**Methods:**

The status of HSP90 and the profiles of the Akt/ mTOR pathway were assessed by immunohistochemistry in 79 samples of UPS, and these data were compared with clinicopathological and histopathological findings. The expressions of indicated proteins were assessed by Western blotting in five frozen samples. After treating UPS cells with the HSP90 inhibitor, we assessed the antitumor effect of the inhibitor.

**Results:**

Immunohistochemically, phosphorylated Akt (p-Akt), p-mTOR, p-S6RP and p-4EBP were positive in 57.3, 51.9, 54.5 and 57.1 % of the UPS samples, respectively. The expressions of those phosphorylated proteins were correlated with each other. HSP90 expression was elevated in 56.4 % of the samples and was correlated with p-Akt, p-mTOR and p-S6RP. The immunohistochemical results were confirmed by Western blotting. The HSP90 inhibitor led to decreased viability and invasiveness of the cells and inactivated the AKT/mTOR pathway *in vitro*.

**Conclusion:**

Elevated expression of HSP90 is a poor-prognosis factor and is involved in the activation of the Akt/mTOR pathway in UPS. HSP90 inhibition is a potential treatment option for UPS.

**Electronic supplementary material:**

The online version of this article (doi:10.1186/s12885-015-1830-8) contains supplementary material, which is available to authorized users.

## Background

Undifferentiated pleomorphic sarcoma (UPS) showing no identifiable line of differentiation is a heterogeneous tumor group as defined by the World Health Organization (WHO) classification [1]. Radiation-induced tumor genesis has also been identified. Heat shock proteins (HSPs) are chaperones responsible for protein folding in normal cells [2], and HSP90, a member of the HSP family, refolds certain denatured proteins under stress conditions and activates these proteins, which are called “client proteins” [3]. The proteins include the growth-stimulating proteins and kinases that support malignant transformation [4].

One of the important client proteins is Akt [3], a serine/threonine kinase activated by phosphoinositide 3-kinase (PI3K). Akt activates the downstream mammalian target of rapamycin (mTOR). The Akt/mTOR pathway plays diverse roles in the normal oncogenic process [5]. In addition to HSP90, another molecule involved in the activation of the Akt/mTOR pathway is phosphatase and tensin homologue (PTEN) [6]. PTEN antagonizes PI3K function, and the loss of PTEN activates the Akt/mTOR pathway. Several studies have demonstrated the activation of the Akt/mTOR pathway in various sarcomas [7–9]. To our knowledge, there is no report of an analysis of the roles of HSP90 and the Akt/mTOR pathway in UPS.

Another signaling pathway that involves HSP90 is the mitogen-activated protein kinase (MAPK) pathway, which plays a key role in the transduction of extracellular signals to cellular responses. There is signaling cross-talk between the AKT/mTOR and MAPK pathways. The MAPK pathway requires the HSP90-chaperone function for proper folding and stability [4]. The relationship between the MAPK pathway and HSP90 in UPS remains to be clarified.

HSP90 inhibitors are well-known molecular therapeutic agents. HSP90 inhibition results in a mechanism-based change in the expression of specific proteins [10]. In terms of the Akt/mTOR pathway, the inhibition of HSP90-Akt binding leads to the dephosphorylation and inactivation of Akt [3]. We postulated that an HSP90 inhibitor might be effective against UPS if an elevated expression of HSP90 is involved in the activation of the Akt/mTOR and MAPK pathways in UPS.

First, we reclassified tumors that had been diagnosed as pleomorphic sarcoma (including unclassified/undifferentiated pleomorphic sarcoma). In these reclassified UPSs, we analyzed the HSP90 expression, Akt/mTOR pathway activation and the relationship between HSP90 expression and Akt/mTOR pathway activation, and we investigated the status of the MAPK pathway. The antitumor effect of an HSP90 inhibitor on UPS cell lines *in vitro* was also evaluated.

## Methods

### Patients and materials

**Fig. 1:**
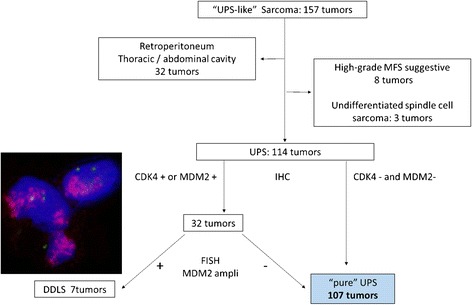
Reclassification of “UPS-like” sarcomas to “pure” UPS. We excluded 32 tumors in the body cavity because the tumors could be a component of a sarcomatoid carcinoma or dedifferentiated liposarcoma (DDLS). Three tumors were reclassified as undifferentiated spindle cell sarcomas. Eight pleomorphic sarcomas with focal myxoid stroma were also excluded because the difference in the diagnosis between UPS with focal myxoid component and high-grade myxofibroarcoma (MFS) was ambiguous. Seven undifferentiated pleomorphic sarcomas with MDM2 amplification were excluded because their biological character is similar to that of DDLS. A FISH analysis showed an MDM2 red signal present in a cluster in a tumor cell nucleus (green: centromere of chromosome 12)

We reassessed individual patients’ 157 tumors (150 primary tumors, 6 recurrent tumors, and 1 metastatic tumor) that had been diagnosed as pleomorphic sarcoma at the Department of Anatomic Pathology, Kyushu University, Fukuoka, Japan between 2000 and 2014, according to the flow chart provided as Fig. 1. Radiation-induced sarcomas or secondary sarcomas after chemotherapy were not included in this study. In each case, we carefully reviewed the hematoxylin and eosin (H&E)-stained slides. We also examined 32 cases that were immunoreactive for CDK4 (Invitrogen, Carlsbad, CA) or MDM2 (Calbiochem, La Jolla, CA) for MDM2 gene amplification by fluorescence in situ hybridization (FISH).

After the reclassification, 107 of the 157 tumors were diagnosed as UPSs. The reassessed diagnosis of UPS was made according to the WHO 2013 classification [1]. We excluded 50 sarcomas, including pleomorphic sarcomas located in the thoracic/abdominal cavity or the retroperitoneum (32 cases), undifferentiated spindle cell sarcomas (3 cases), pleomorphic sarcomas with focal myxoid stroma (8 cases), and undifferentiated pleomorphic sarcomas with MDM2 gene amplification (7 cases). Follow-up information was available in 102 tumor cases. The median follow-up period after surgery was 36 months (range 3–168 months), excluding the cases of the patients who had died.

We evaluated the extent of necrosis and mitosis according to the French Federation of Cancer Centers (FNCLCC) grading system [11]. The seventh edition of the American Joint Committee on Cancer (AJCC) staging system was applied to each case [12]. The Institutional Review Board at Kyushu University approved this study (permission code 25–79). Written informed consent for participation in the study was obtained from the patients or from a parent of pediatric patients.

### Cell culture and reagents

The human UPS cell lines FPS-1 and FU-MFH-2 were cultured in RPMI-1640 medium and Dulbecco’s modified Eagle’s medium (DMEM)/F-12 [13, 14]. These media preparations were supplemented with 10 % fetal bovine serum (FBS) plus penicillin and streptomycin. The HSP90 inhibitor alvespimycin (17-dimethylaminoethylamino- 17-demethoxygeldanamycin; 17-DMAG) was purchased from Seleck Chemicals (Houston, TX) and diluted in dimethyl sulfoxide (DMSO).

### Immunohistochemistry (IHC)

Immunohistochemical staining was performed as described [9]. Among the 107 UPSs, 79 formalin-fixed paraffin-embedded samples (74 primary tumors, 4 recurrent tumors, and 1 metastatic tumor) were available for this IHC analysis. Antigen retrieval was performed by boiling the slides with 10 mM sodium citrate (pH 6.0) or Target Retrieval Solution (Dako, Carpinteria, CA). We used rabbit antibodies for phosphorylated (p) Akt (p-Akt) (serine 473 [Ser473]; 1:50 dilution), p-mTOR (Ser2448; 1:100 dilution), p-S6 (Ser235/236; 1:100 dilution), p-4E-BP1 (threonine 37/46 [Thr37/46]; 1:400 dilution), p- mitogen-activated protein kinase1/2 (p-MEK1/2) (Ser217/221; 1:100 dilution), p-extracellular signal-regulated kinase (p-ERK1/2) (Thr202/Tyr204; 1:400 dilution), PTEN (1:50 dilution) and HSP90 (1:400 dilution) (Cell Signaling Technology, Danvers, MA). The mouse antibody for Ki-67 (MIB-1) (1:100 dilution) (Dako) was used as the primary antibody.

The results for p-Akt, p-mTOR, p-S6RP, p-4E-BP, p-MEK1/2, p-ERK1/2 and PTEN were evaluated according to the method of Dobashi et al. [15]. When >10 % of the tumor cells showed nuclear and/or cytoplasmic staining with stronger intensity than the endothelial cells, the samples were judged as positive. We also assessed the HSP90 expression as described by Song et al. [16]. Cases with >5 % nuclear- or cytoplasmic-positive tumor cells with stronger intensity than the endothelial cells were classified as positive. The MIB-1 labeling index was defined as the percentage of immunoreactive cells divided by the total number of cells in the evaluated area. Five viable fields from the area of maximal labeling were chosen for counting. Each section was evaluated independently by three investigators.

### Fluorescence in situ hybridization (FISH)

To rule out dedifferentiated liposarcoma (DDLS), we examined 32 clinical samples and the UPS cell lines (FPS-1 and FU-MFH-2) for MDM2 gene amplification. Fluorescence in situ hybridization (FISH) using the MDM2 (TexRed)/CEN1q (FITC) Dual Color FISH Probe (Abnova, Taipei, Taiwan) was performed on tissue sections. Each formalin-fixed paraffin-embedded tissue was cut at 4-μm thickness. The deparaffinization, pretreatment, and protease digestion procedures followed the manufacturer’s protocol. The probe cocktail labels the human chromosomal region MDM2 with a red signal and the centromeric region of chromosome 12 (12p11.12 sequences) with a green signal. We counted the signals under a microscope (BX53, Olympus, Tokyo) and analyzed them with the cellSens Standard software (version 1.9; Olympus). A minimum of 20 nuclei per slide were visualized. Amplification was defined as >2.0 fluorescent signals per cell [17].

### Western blotting

The Western blot analysis was conducted as described [18]. Protein was extracted from five available frozen samples paired with normal muscular tissue and from the cultured UPS cells after they were treated with 17-DMAG (0.3 μmol/L) for 6, 12 or 24 h. In addition to the antibodies used for the IHC analysis, the rabbit antibodies for pan-Akt (C67E7; 1:400 dilution), pan-mTOR (1:400 dilution), and pan-S6RP (5G10; 1:400 dilution) (Cell Signaling Technology) were used as the primary antibody. For an internal control, an anti-glyceraldehyde 3-phosphate dehydrogenase (anti-GAPDH) (1:5,000 dilution; Santa Cruz Biotechnology, Santa Cruz, CA) mouse monoclonal antibody was used. For p-MEK1/2 and p-Erk1/2, the comparison of protein expression between tumor tissue and normal muscular tissue was not made because the MAPK pathway may be activated under a state of perioperative stress in skeletal muscle [19].

The phosphorylation scores were calculated using the formula reported by Setsu et al. as follows: (p-protein [tumor]/pan-protein [tumor])/(p-protein [normal]/pan-protein [normal]) [20]. This formula was applied to Akt, mTOR and S6RP. The intensities of p-4EBP, PTEN and HSP90 were compared with that of GAPDH instead of pan-protein.

### Cell growth assay

Tumor cells were harvested at 70 % confluence, seeded at 3 × 10^3^ cells per well in 96-well plates, and incubated in the medium for 12 h. 17-DMAG was added to each well at the indicated concentration, and the incubation was continued for another 48 or 72 h as described by Mayer et al. [21]. Viability was assessed by performing a WST-8 assay using the Cell Counting Kit 8 (CCK-8, Dojindo Molecular Technologies, Kumamoto, Japan) according to the manufacturer’s protocol. The absorbance at 450 nm was measured by a microplate reader (Model 680 Microplate Reader, Bio-Rad Laboratories, Hercules, CA). The percentage growth was calculated relative to untreated controls. Each assay was carried out in triplicate, with results based on three independent experiments.

### Wound-healing assay

A wound-healing assay was conducted using the UPS cell lines FPS-1 and FU-MFH-2. Confluent cell monolayers in 6-well plates were wounded by scraping with a micropipette tip. The wells were treated with 0.1 nmol/L of 17-DMAG. The cell motility was assessed by comparing the sizes of the scratches at 0 h and at 12 h with a microscope (BZ-8000, Keyence, Tokyo). Each assay was conducted in triplicate and repeated three times.

### Matrigel invasion assay

Cell invasiveness was assessed using the 24-well Biocoat Matrigel invasion chamber (BD Biosciences, San Diego, CA) according to the manufacturer’s protocol. Cells (FPS-1 and FU-MFH-2) were detached from culture plates and resuspended in the upper chamber separated by an 8-mm pore-size filter at 1 × 10^5^ per chamber in serum-free media. Outer wells were filled with media containing 5 % FBS. The chambers were treated with the 0.1 nmol/L of 17-DMAG. The cells were incubated at 37 °C with 5 % carbon dioxide for 24 h, and then non-invading cells were removed by wiping with a cotton swab. Invading or migrating cancer cells were fixed to the lower surface of the transwell membrane with 70 % ethanol, stained with H&E, and counted in five random fields at 200× magnification.

The membrane was mounted on a microscope slide, and migrated cells were counted in five random high-power fields. Data are expressed as the percentage of invasion through the Matrigel matrix and membrane relative to the migration through the control membrane, according to the manufacturer’s manual.

### Statistical analysis

Continuous variables are presented as mean ± standard deviation values. All parameters were analyzed for their correlation to one another by using the Fisher exact test. The survival correlations are illustrated with Kaplan-Meier curves using the cutoff at 15 years, and survival analyses were performed using the log-rank test. In the multivariate analysis, a Cox proportional hazards model was used to examine risk factors picked up in the univariate analysis for clinicopathological parameters and the immunohistochemical results. *In vitro* data were analyzed by Student’s *t*-test. A two-sided *p*-value <0.05 was considered significant. The data analyses were conducted with the JMP statistical software package (version 9.0.2; SAS Institute, Cary, NC).

## Results

### The AJCC stage was identified as a poor-prognosis risk factor based on the clinicopathological findings after reclassification

After the classification, 107 (102 primary, 4 recurrent, and 1 metastatic) of the 157 tumors (68 %) were diagnosed as UPS. The 107 tumors were located in the extremities in 65 cases (thigh, 40; upper arm, 11; lower leg, 9; and forearm, 5), in the trunk wall in 31 cases (buttock 13, back 7, shoulder 5, chest wall 4, and abdominal wall 2) and in the head and neck in 11 cases. We excluded a total of 50 tumors from the study. Thirty-two tumors in the thoracic/abdominal cavity were excluded because of the possibility that the tumor could be a component of sarcomatoid carcinoma or DDLS [22]. Three tumors were reclassified as undifferentiated spindle cell sarcomas, because pleomorphic cells were inconspicuous. Eight pleomorphic sarcomas with focal myxoid stroma were also excluded because the difference in the diagnosis between UPS with focal myxoid component and high-grade myxofibrosarcoma (MFS) seemed ambiguous. Seven of the 32 (21.9 %) undifferentiated pleomorphic sarcomas with MDM2 amplification were excluded because their biological character is similar to that of DDLS [23]. The amplification of the *MDM2* gene locus is illustrated in Fig. 1.

**Fig. 2:**
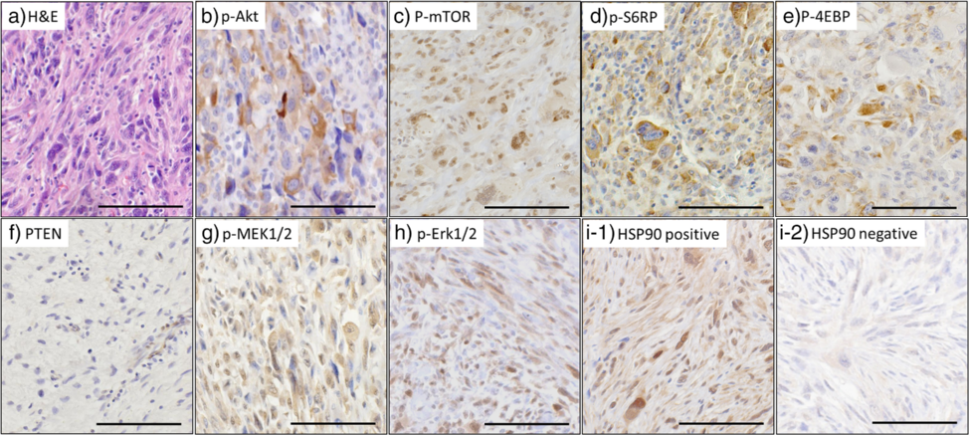
Representative H&E stain (**a**) and immunohistochemical staining (**b**–**g**). Positive stains for p-Akt (**b**), p-mTOR (**c**), p-S6RP (**d**), and p-4EBP (**e**) in the Akt/mTOR pathway and p-MEK1/2 (**f**), and p-Erk1/2 (**g)** in the MAPK pathway. Decreased expression of PTEN in the tumor cells and immunostaining of endothelial cells (**h**). Immunohistochemical staining with different intensities for HSP90 (**i**). Immunohistochemically nuclear and/or cytoplasmic staining was judged as positive. Bar: 100 μm

The data of the 102 primary tumors are summarized in Table 1. Representative H&E staining is shown in Fig. 2. In the univariate analysis, overall survival (OS) was significantly related to differences in large (>50 mm) tumor size, deep location, the existence of metastasis and tumor necrosis, more frequent mitosis, FNCLCC grade 3, and high AJCC stage (i.e., III or IV). As for event-free survival (EFS), the prognostic risk factors were tumor size >50 mm, deep location, the existence of tumor necrosis, frequent mitosis with ≥ 20 per 10 high-power fields (HPF), FNCLCC grade 3, and higher AJCC stage (IV > III > II). The multivariate analysis demonstrated that the AJCC stage was a poor-prognosis risk factor for OS and EFS. Tumor size, the existence of metastasis and tumor necrosis, mitotic activity, and FNCLCC grade were excluded from this multivariate analysis, because the AJCC stage was determined by these parameters.

**Table 1:** Clinicopathological parameters and survival analysis in 102 primary UPS tumors

					*P*	
Variable	Group	No.	(%)	Analyzed groups	Overall survival	Event-free survival
Sex	Male	45	44.1		0.3077	0.1815
	Female	57	55.9			
Age (17 ~ 93, mean: 62)	<62	53	52		0.3714	0.1518
	≧62	49	48			
Size	<5 cm	35	34.3		0.0058^a^	0.0008^a^
	≧5 cm	56	54.9			
	N/A	11	10.8			
Location	Superficial	42	41.2		0.0154^a^	0.0193^a^
	Deep	54	52.9			
	NA	6	5.9			
Chemotherapy	Yes	20	19.6		0.5761	0.9270
	No	30	29.4			
	NA	52	51.0			
Surgical margin	Positive	18	17.6		0.3069	0.9937
	Negative	45	44.1			
	NA	39	38.3			
Metastasis	+	28	27.5		<0.0001^a^	<0.0001^a^
	-	74	72.5			
Reccurence	+	16	15.7		0.9212	<0.0001^a^
	-	86	84.3			
Necrosis	0, score 0	58	56.9	Score 0 vs others	0.0011^a^	0.0101^a^
	<50 %, score 1	31	30.4	Score 1 vs score 2	0.464	0.6221
	≧50 %, score 2	13	12.7	Score 2 vs others	0.0344^a^	0.2112
Mitosis	0–9/10 HPF, score 1	53	52	Score 1 vs others	0.0003^a^	0.0005^a^
	10–19/10 HPF, score 2	28	27.4	Score 2 vs score 3	0.0291^a^	0.4872
	20≧/10 HPF, score 3	21	20.6	Score 3 vs others	<0.0001^a^	0.0077^a^
MIB-1 LI	<10 %	20	26.7		0.0569	0.0533
	≧10 %	50	66.7			
	NA	5	6.6			
FNCLCC	2	64	62.7		<0.0001^a^	00002^a^
	3	38	37.3			
AJCC 7th ed	II	59	57.8	Stage II vs others	<0.0001^a^	<0.0001^a^
	III	15	14.7	Stage III vs IV	0.0614	<0.0001^a^
	IV	28	27.5			

*AJCC* American Joint Committee on Cancer, *FNCLCC* French Federation of Cancer Centers, *HPF* high-power fields, *LI* labeling index, *N/A* not available, *No*. number of patients. The Fisher exact test or the log-rank test was used if not indicated otherwise

^a^Statistically significant

### The Akt/mTOR pathway and HSP90 were immunohistochemically correlated and revealed to be risk factors for a poor prognosis in UPS

The IHC results for the Akt/mTOR pathway, the MAPK pathway and HSP90 are illustrated in Fig. 2. The correlation between the IHC results and clinicopathological data are summarized in Additional file 1: Table S1. The positive ratios were as follows: p-Akt, 57.3 %; p-mTOR, 51.9 %; p-S6RP, 54.5 %; p-4EBP, 57.1 %; p-MEK1/2, 48.6 %; p-ERK1/2, 74.0 %; PTEN, 77.3 %; and HSP90, 56.4 %. The cases in which endothelial cells failed to reveal any staining were excluded from the evaluation: p-Akt, four cases; p-mTOR, two; p-S6RP, two; p-4EBP, two; p-SMEK1/2, two; p-Erk1/2, four; and PTEN, three cases.

The positivities for p-Akt, p-mTOR and p-S6RP and p-4EBP were significantly correlated with each other (*p* < 0.05). The elevated expression of HSP90 showed significant correlations with the positivities for p-Akt (*p* = 0.0003), p-mTOR (*p* = 0.0223), and p-S6RP (*p* = 0.0004). The loss of PTEN was not correlated with the activation of the Akt/mTOR pathway or the elevated expression of HSP90. The positivities for p-MEK1/2 and p-Erk1/2 were significantly correlated with each other (*p* = 0.021). The positivity for p-MEK1/2 was correlated with the elevated expression of HSP90 (*p* = 0.0195).

Clinicopathologically, the positivity for p-mTOR was correlated with the existence of metastasis (*p* = 0.003), tumor necrosis (*p* = 0.0007) and >10 % MIB-1 labeling index (LI) (*p* = 0.0038). p-S6RP was correlated with the existence of metastasis (*p* = 0.0057), tumor necrosis (*p* = 0.0189), frequent mitosis (*p* = 0.0377) and higher AJCC stage (*p* = 0.0158). p-4EBP was correlated with large tumor size (*p* = 0.0316), the existence of metastasis (*p* = 0.0014), >10 % MIB-1 LI (*p* = 0.0095), and higher AJCC stage (*p* = 0.0173). HSP90 was correlated with older (>62 years) patient age (*p* = 0.034), frequent mitosis (*p* = 0.0235) and higher AJCC stage (*p* = 0.0342).

**Fig. 3:**
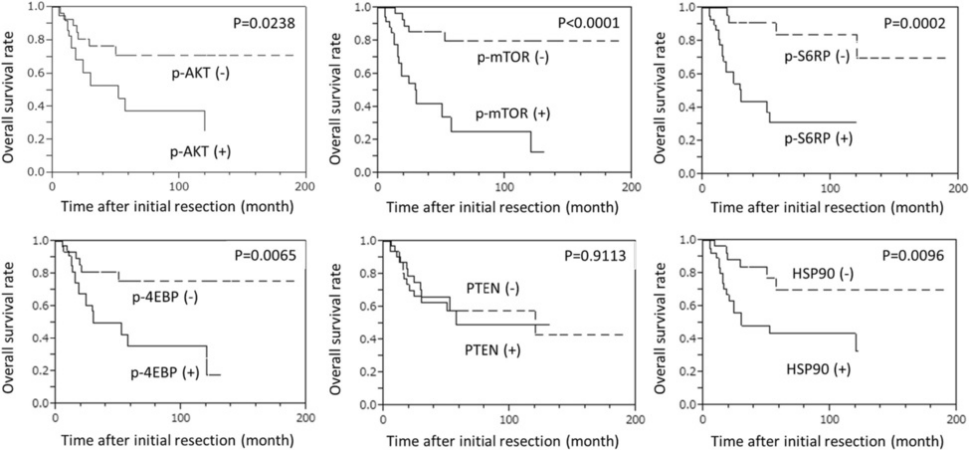
Kaplan-Meier survival curves for overall survival (OS) according to the results of the IHC analysis. Positivities for p-Akt, p-mTOR, p-S6RP, p-4EBP and HSP90 were correlated with OS by log-rank test. PTEN expression was not correlated with OS

In the univariate analysis, the positivities for p-Akt, p-mTOR, p-S6RP and p-4EBP and elevated HSP90 expression were significant risk factors for a poor prognosis. The Kaplan-Meier survival curves for OS according to the IHC results are illustrated in Fig. 3. The multivariate analysis for immunohistochemical parameters adjusted by AJCC stage and tumor location indicated that the positivities for p-Akt, p-mTOR, p-S6RP and HSP90 were poor prognostic factors for OS (Additional file 2: Table S2).

### The Western blotting showed that the level of HSP90 expression in the tumor tissues was higher than that in the normal tissues

The Western blotting results are illustrated in Additional file 3: Figure S1. p-Akt, p-mTOR, p-S6RP and p-4EBP were detected in all tumor samples. The densitometric analysis demonstrated that immunohistochemically positive proteins showed higher (>1.0) P-scores, which indicated that the tumor was phosphorylated at a higher level compared to non-neoplastic tissue. As for PTEN and HSP90, the expression levels in Western blotting comparatively corresponded with the immunohistochemical results. The level of HSP90 expression in the tumor tissues was higher than that in the normal tissues.

### 17-DMAG caused a decrease in the viability and invasiveness of the cells and a blockage of the Akt/ mTOR pathway in UPS

Both cell lines showed no amplification of the *MDM2* gene locus by FISH analysis.

**Fig. 4:**
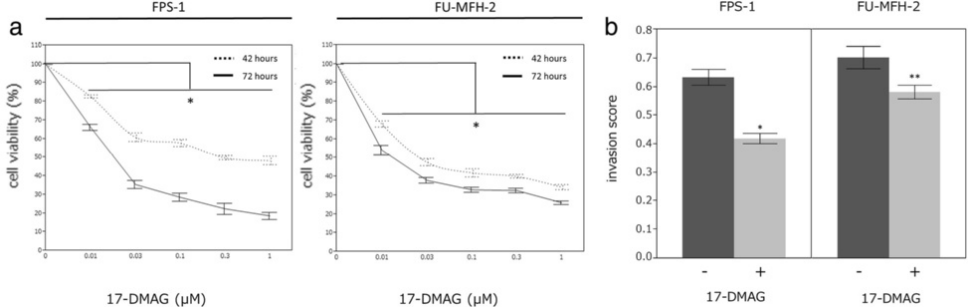
17-DMAG suppressed the growth of UPS cells and caused a decrease in the invasion of cells. (**a**) HSP90 inhibition decreased the viability in a dose- and time-dependent manner. (**b**) The Matrigel invasion assay showed that 17-DMAG caused a decrease in the invasion of both cell lines. Error bars = standard deviation.* *p* < 0.01, ** *p* < 0.05

**Fig. 5:**
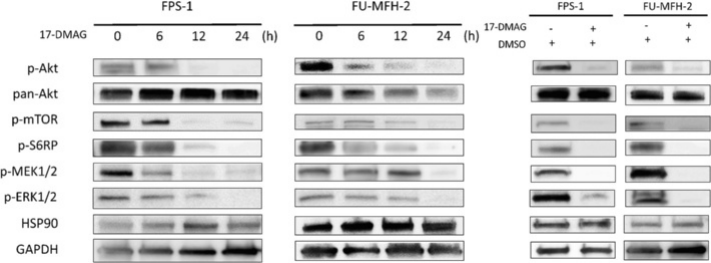
17-DMAG reduced the protein expression of the Akt/mTOR pathway. Cell lines were treated with 17-DMAG (0.3 μmol/L) for 6, 12 and 24 h. The 17-DMAG**-**induced changes in the activation status of the AKT/mTOR and MAPK pathways were evaluated by Western blotting. Decreased p-Akt, p-mTOR, p-S6RP, p-MEK1/2 and p-ERK1/2 expressions were observed in both cell lines. The HSP90 expressions showed no clear difference. The cell lines were also treated with or without 17-DMAG (0.3 μmol/L) diluted in DMSO for 24 h. DMSO had no influence on the status of the AKT/mTOR or MAPK pathways

The effects of 17-DMAG on the two UPS cell lines are illustrated in Figs. 4 and 5 and Additional file 4: Figure S2. HSP90 inhibition significantly decreased the viability of the FPS-1 and FU-MFH-2 cells in a dose- and time-dependent manner (*p* < 0.01) (Fig. 4a). In the Matrigel invasion assay, 17-DMAG caused a decrease in the invasiveness of both cell lines, FPS-1 (*p* = 0.009) and FU-MFH-2 (*p* = 0.041) (Fig. 4b). In the wound-healing assay, 17-DMAG had no influence on the cell motility in either cell line (Additional file 4: Figure S2).

The Western blotting showed decreased expressions of p-Akt, p-mTOR, p-S6RP, p-MEK1/2 and p-ERK1/2 in both cell lines (Fig. 5). The expression of HSP90 showed no clear change. The p-Akt expression was decreased at 6 h and at 12 h after the treatment with 17-DMAG, and the p-mTOR and p-S6RP expressions were decreased at 12 h. This sequential alternation indicated that the inhibition of HSP90-Akt binding blocked the Akt/ mTOR signaling pathway.

## Discussion

The purpose of the present study was to determine whether HSP90 could be a therapeutic target against UPS. We carefully selected the UPSs and observed elevated HSP90 expression and AKT/mTOR pathway activation by immunohistochemistry and Western blotting. The multivariate analysis indicated that the positivities for p-Akt, p-mTOR, p-S6RP and HSP90 were independent poor-prognosis factors. HSP90 inhibition decreased the viability and invasiveness of UPS cells and inactivated the AKT/mTOR pathway *in vitro*.

To conduct our study of UPS, we reclassified tumors that had been diagnosed as pleomorphic sarcoma. The basic principle for the diagnosis of soft tissue sarcomas is to find the specific line of differentiation and not to throw tumors into the “wastebasket” of UPS [24, 25]. However, the difference in the morphology remains to be clarified between UPS with myxoid stroma and a high-grade component of MFS or between UPS in the body cavity and sarcomatoid carcinoma [22]. Moreover, a FISH study is sometimes required for the diagnosis in addition to the morphology findings.

Amplification of MDM2, the driving genes in 12q13-15, can frequently be detected by FISH [26, 27]. Representative sarcomas with adipocytic differentiation that show MDM2 gene amplifications are defined as DDLS. However, a recent study reported that peripheral UPS with MDM2 gene amplification is considered DDLS even if there is no well-differentiated liposarcoma area [23]. We retrieved definitive UPSs, and several poor prognostic factors were identified including large (>50 mm) tumor size, deep location, the existence of tumor necrosis and frequent mitosis. The AJCC stage was an independent poor-prognosis risk factor for overall survival and event-free survival in the multivariate analysis, supporting the usefulness of the FNCLCC grades and AJCC staging system.

In clinical samples of reclassified UPSs, we analyzed the HSP90 expression and Akt/mTOR pathway activation by immunohistochemistry and Western blotting. HSP90 is a chaperone interacting with client proteins that are essential for constitutive cell signaling and adaptive response induced by stress [28]. HSP90 also implicates client proteins in activated oncogenesis [29, 30]. It was revealed that Hsp90 is up-regulated in tumor cells and transformed cells, and Hsp90 expression was elevated in clinical samples of breast cancer and cholangiocarcinoma [16, 31, 32]. The present study’s findings demonstrated that the HSP90 expression was elevated in approx. 50 % of the UPSs, and this elevated expression was an independent poor-prognosis factor in UPS in the multivariate analysis. The elevated expression of HSP90 was significantly correlated with other poor-prognosis factors including mitotic figure, FNCLCC grade and AJCC stage. In addition, the Western blotting analysis showed that the level of HSP90 expression in the tumor tissues was higher than that in the non-neoplastic tissue, although the number of frozen samples was small. This result suggests that HSP90 could be a candidate target molecule in the treatment of UPS.

Akt is a serine/threonine kinase activated by PI3K, and activated AKT initiates a cascade of downstream signaling [33]. The Akt/ mTOR pathway modulates cellular function in response to extracellular signals and can lead to tumor initiation and progression [34, 35]. Our group reported that this pathway was activated in leiomyosarcomas and malignant peripheral nerve sheath tumors in approx. 70 and 50 % of the cases, respectively [7, 20]. Lahat et al*.* reported that 20 % of human UPSs showed strong expression of p-Akt [36]. The present study’s findings demonstrated that Akt and downstream molecules were activated in approx. 50 % of the UPSs. This discrepancy may have occurred because the diagnostic criteria for UPS were revised by WHO2013 after the previous report had been published [1, 36]. In our study, positivities for p-Akt, p-mTOR and p-S6RP were independent poor-prognosis factors in the multivariate analysis, and they were significantly correlated with the elevated expression of HSP90 and clinicopathological poor-prognosis factors. These data suggest that the inhibition of the Akt/mTOR pathway could have therapeutic benefit for the treatment of UPS.

Abnormalities of several molecules could be responsible for the activation of the Akt/mTOR pathway. Some studies have revealed that gene mutations in PI3KCA or AKT1 activate the Akt/mTOR pathway [37, 38]. As of this writing, no gene mutations have been detected in UPS, to our knowledge. Another molecule involved in the pathway is PTEN. PTEN antagonizes PI3K function, and the loss of PTEN increases PI3K-AKT signaling [6]. In the present study, we investigated the status of PTEN in UPSs, and our immunohistochemical analysis showed that the loss of PTEN was not correlated with the activation of the Akt/mTOR pathway.

On the basis of the results of our analysis of clinical samples, we investigated the effect of an HSP90 inhibitor on two UPS cell lines. Among biomolecular targets, the Akt/mTOR pathway presents a well-known target for molecular therapeutics [39]. A recent large randomized phase III trial evaluated the effects of an mTOR inhibitor against metastatic sarcomas [40]. However, the result of this trial was discouraging. This may have occurred because there is cross-talk between the AKT/mTOR and MAPK pathways, and MAPK pathway signaling can be enhanced by mTOR inhibition [41, 42]. Therefore, targeting the Akt/mTOR pathway without enhancing the MAPK pathway seemed to be more preferable than using a single-agent mTOR inhibitor in chemotherapy. In our immunohistochemical study, the elevated expression of HSP90 was significantly correlated with the activation of the Akt/mTOR pathway and tended to be correlated with the activation of the MAPK pathway. This result suggests that an HSP90 inhibitor could lead to the blockade of the Akt/mTOR and MAPK pathways in UPS.

HSP90 inhibition leads to a mechanism-based change in the expression of specific proteins [4]. HSP90 protects phosphorylated Akt from dephosphorylation, and the inhibition of Akt-Hsp90 binding leads to the dephosphorylation and inactivation of Akt [3, 43]. The MAPK pathway also requires the HSP90-chaperone function for proper folding and stability [4]. Although HSP90 is expressed in normal cells, HSP90 derived from tumor cells has a 100-fold higher binding affinity for HSP90 inhibitor compared to HSP90 derived from normal cells, which indicates the selectivity of HSP90 inhibitors toward tumor cells [44].

We selected 17-DMAG among the several HSP90 inhibitors, because 17-DMAG has already been evaluated in clinical trials for patients with ovarian and breast cancer [45, 46]. Our *in vitro* study yielded evidence that 17-DMAG decreased the viability and the invasiveness of UPS cells, and 17-DMAG inactivated the kinase activity of the Akt/mTOR and MAPK pathways although the cell motility remained unchanged. Activation of the Akt/mTOR pathway contributes to increased cell invasiveness in prostate cancer and breast cancer [47, 48]. Our present findings indicate that an Hsp90 inhibitor would inactivate the Akt-mTOR pathway and subsequently decrease the cell invasiveness in UPS. We did not verify the immediate cause of decreased invasiveness induced by 17-DMAG. Matrix lysis is one of the sequence of biochemical events during tumor cell invasion, and HSP90 is required for the matrix lysis [49]. An investigation of the molecular markers involved in the matrix lysis such as matrix metalloproteinase (MMP) could lead to a better understanding of our present findings. Nevertheless, the HSP90 inhibitor 17-DMAG seems to have a practical application in targeted therapy against UPS compared with an mTOR inhibitor.

## Conclusion

In summary, we conclude that an HSP90 inhibitor could be a potential treatment option against UPS based on our clinicopathological assessment, immunohistochemistry, Western blotting and an *in vitro* study using 17-DMAG on UPS cell lines. Further studies may lead to the realization of an HSP90 inhibitor in a targeted therapy for UPS.

## Additional files

Additional file 1: Table S1. Multivariate Survival Analysis of Immunohistochemical Parameters. CI, confidence interval; HR, hazard ratio; other abbreviations are explained in the Table S2 footnote. a Adjusted by AJCC stage (II vs. III, IV) and tumor location. b *p* < 0.05. (XLSX 10 kb) Additional file 2: Table S2. Immunohistochemical Results and Their Statistical Analysis. EFS, event-free survival; LI, labeling index; OS, overall survival; pAkt, phosphorylated protein kinase B; p-ERK1/2, phosphorylated-extracellular signal-regulated kinase1/2; p-4EBP, phosphorylated eukaryotic translation initiation factor 4E-binding protein; p-MEK1/2, phosphorylated mitogen-activated protein kinase1/2; p-mTOR, phosphorylated mammalian target of rapamycin; p-S6RP, phosphorylated S6 ribosomal protein; PTEN, phosphatase and tensin homologue. The Fisher exact test or the log-rank test was used if not indicated otherwise. a: *p* < 0.05. b: Comparison of no necrosis and presence of necrosis. c: Comparison of 0–9/10 HPF and 10/10 HPF. d: Comparison of stage II and stage III or IV. (XLSX 12 kb) Additional file 3: Figure S1. Protein expression analysis by Western blot analysis. p-Akt, p-mTOR, p-S6RP and p-4EBP were detected in all tumor samples. Immunohistochemically positive proteins showed higher P-score >1.0. As for PTEN and HSP90, the expression levels in Western blotting comparatively corresponded with the immunohistochemical results. T: tumor tissue; N: normal tissue. (PDF 175 kb) Additional file 4: Figure S2. Wound-healing assay showed 17-DMAG had no influence on the cell motility in both cell lines. (PDF 50 kb)

## Abbreviations

AJCC: American Joint Committee on Cancer
DDLS: dedifferentiated liposarcoma
EFS: event-free survival
FISH: fluorescence in situ hybridization
FNCLCC: French Federation of Cancer Centers
HPF: high-power fields
LI: labeling index
OS: overall survival
pAkt: phosphorylated protein kinase B
p-ERK1/2: phosphorylated-extracellular signal-regulated kinase1/2
p-4EBP: phosphorylated eukaryotic translation initiation factor 4E-binding protein
p-MEK1/2: phosphorylated mitogen-activated protein kinase1/2
p-mTOR: phosphorylated mammalian target of rapamycin
p-S6RP: phosphorylated S6 ribosomal protein
PTEN: phosphatase and tensin homologue
UPS: undifferentiated pleomorphic sarcoma
WHO: World Health Organization
