# Supplementary material for: Elevated expression of HSP90 and the antitumor effect of an HSP90 inhibitor via inactivation of the Akt/mTOR pathway in undifferentiated pleomorphic sarcoma
Source: BMC Cancer. 2015 Oct 26;15:804. doi: 10.1186/s12885-015-1830-8 (PMC4623920; doi:10.1186/s12885-015-1830-8)
Supplement: Additional file 3: Figure S1. — Protein expression analysis by Western blot analysis. p-Akt, p-mTOR, p-S6RP and p-4EBP were detected in all tumor samples. Immunohistochemically positive proteins showed higher P-score >1.0. As for PTEN and HSP90, the expression levels in Western blotting comparatively corresponded with the immunohistochemical results. T: tumor tissue; N: normal tissue. (PDF 175 kb) [file 12885_2015_1830_MOESM3_ESM.pdf]

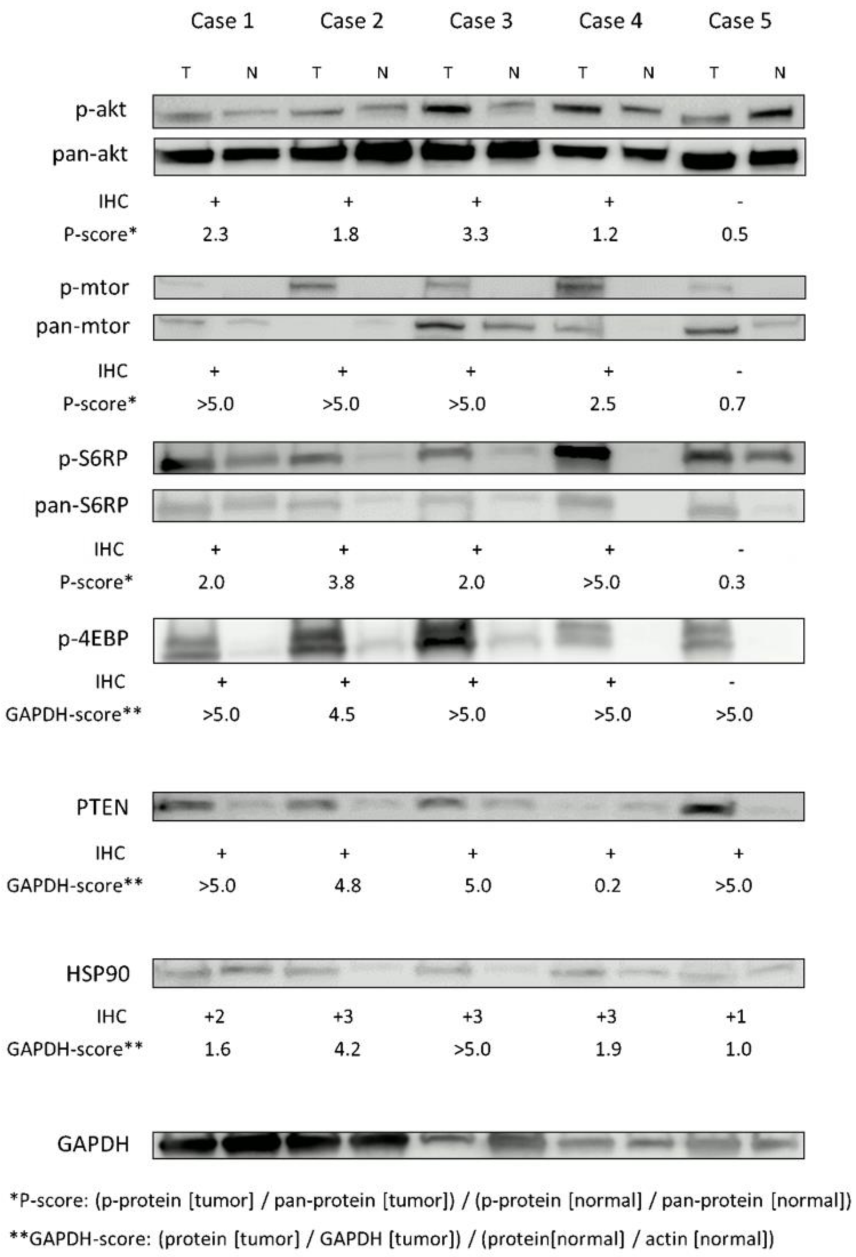

Additional Fig. 1. Protein expression analysis by Western blot analysis. p-Akt, p-mTOR, p-S6RP and p-4EBP were detected in all tumor samples. Immunohistochemically positive proteins showed higher P-score >1.0. As for PTEN and HSP90, the expression levels in Western blotting comparatively corresponded with the immunohistochemical results. T: tumor tissue; N: normal tissue.
