# Supplementary material for: Elevated expression of HSP90 and the antitumor effect of an HSP90 inhibitor via inactivation of the Akt/mTOR pathway in undifferentiated pleomorphic sarcoma
Source: BMC Cancer. 2015 Oct 26;15:804. doi: 10.1186/s12885-015-1830-8 (PMC4623920; doi:10.1186/s12885-015-1830-8)
Supplement: Additional file 4: Figure S2. — Wound-healing assay showed 17-DMAG had no influence on the cell motility in both cell lines. (PDF 50 kb) [file 12885_2015_1830_MOESM4_ESM.pdf]

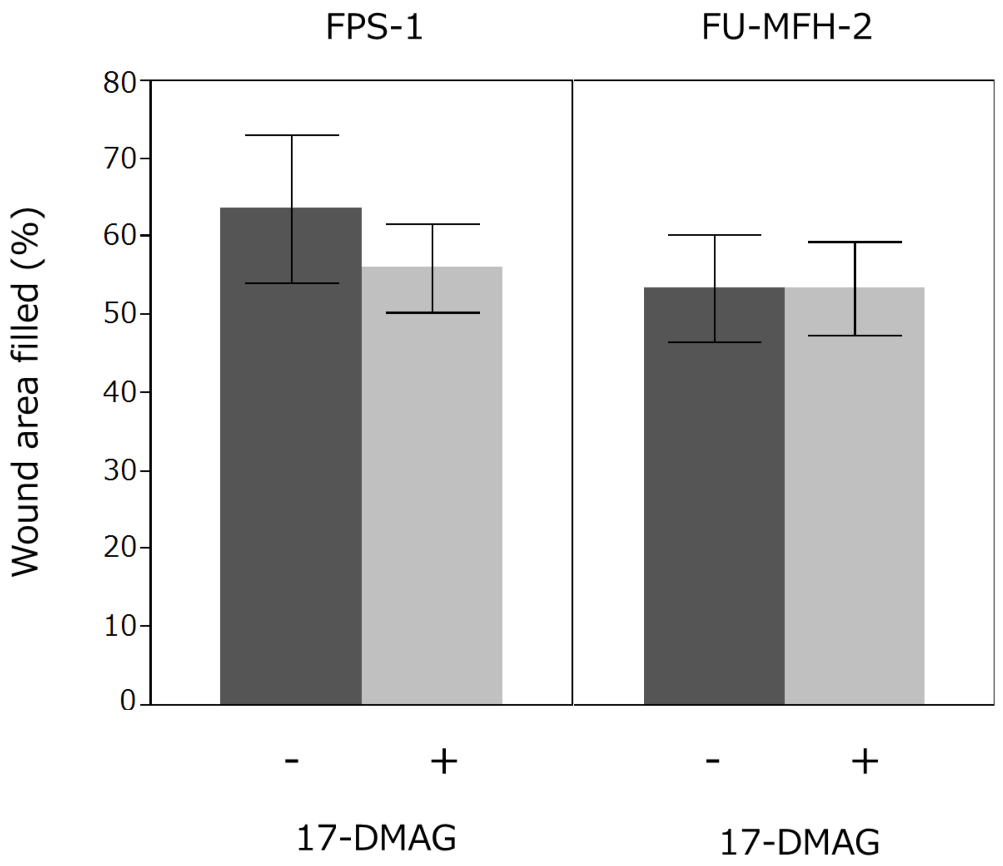

Additional Fig. 2 Wound-healing assay showed 17-DMAG had no influence on the cell motility in both cell lines.
